# Changing visions in ESP development and teaching: Past, present, and future vistas

**DOI:** 10.3389/fpsyg.2023.1140659

**Published:** 2023-04-17

**Authors:** An Qi Dou, Swee Heng Chan, Min Thein Win

**Affiliations:** ^1^School of Education, Taylor’s University, Kuala Lumpur, Malaysia; ^2^School of Medicine, Taylor’s University, Kuala Lumpur, Malaysia

**Keywords:** ESP development, needs analysis, the learning and teaching of ESP, semi-systematic review, future of ESP

## Abstract

Globalization and international development in language education have inspired a shift from the learning of traditional College English to English for Specific Purposes (ESP). This article begins with a section on the methodology used to develop the literature review. From various literatures, a historical perspective was first presented for the period, 1962 to the present day, and accompanied by a review on the teaching approaches. The purpose was to reveal emerging trends in ESP development and forefront the strength of association between ESP development and the changes in teaching approaches. Then it focuses on the relationship between needs analysis and ESP, as needs analysis is well recognized as a vital ESP characteristic and it is given a comprehensive revisit as an update in ESP development. The review continues with some insights into recent studies from various countries to reflect on various aspectual developments of current ESP practices that illustrate the dynamics of growing research agendas that have implications for current and future ESP research directions. Finally, future vistas for ESP development and teaching are affirmed. The paper concludes on the note on the importance of knowing past and future ESP developments, and the prioritizing of effective teaching based on soundly designed materials tailored to particular student-centered needs and wants.

## 1. Introduction

Traditionally, when educators and applied linguists discussed the concept of teaching of EFL (English as a foreign language), they referred to the teaching of what is known today as general English. However, this understanding has changed over the past few decades as the teaching of ESP gained popularity, especially in non-native English-speaking countries. A change in teaching methods was also needed to maximize the educational or learning outcomes of the foreign language teaching process that tends to lean toward the learning of ESP. This includes the design of specific educational learning objectives for short-term or long-term courses, or programs to fulfill the following: (1) the specific needs of the learners, (2) the overall goals of the course or program, and (3) the specific type of training learners hope to achieve upon completion of the program (Dudley-Evans and Saint John, 1998; Hyland, 2006). Ultimately, the goal of all ESP programs is to help learners become more adept in the use of language aligned to their specific disciplines or professional lives (Abuklaish, 2014).

An important characteristic of an ESP course is that the materials and goals are tailored to the learners’ specific needs. They would concentrate on the language, identified skills, and genres that are most relevant to the specific activities that learners need to perform in so as to use English efficiently. In the words of Johns (2012), the teaching of ESP involves pursuing the following key concerns:

*How to identify learner needs, the nature of the genres that learners need to be able to produce as well as participate in, and how to know if our learners have been able to do this successfully, and if not, what we can do to help them (p. 7).*

Belcher (2009) describes the development of ESP as having several branches and sub-branches. It includes English for Academic Purposes (EAP), branching off into English for General Academic Purposes (EGAP), English for Specific Academic Purposes (ESAP) and English for Occupational Purposes (EOP). Sub branches include English for Vocational Purposes (EVP), English for Medical Purposes (EMP), English for Business Purposes (EBP), English for Legal Purposes (ELP) and English for Science and Technology (West, 1994). Today, we see that many more subbranches have evolved, illustrating the vibrant demands of various stakeholders who see the value of learning ESP in accordance with the specific needs associated with various work disciplines as they gain great significance in tandem with developing economies worldwide.

## 2. Methodology of the literature review

A semi-systematic literature review (LR) approach is used to meet the needs of this review. The general aim of the LR is to identify gaps in research and also to engage with the ESP theory development, and to parallel the developments with the emergence of discernible teaching approaches. A semi-systematic review approach was adopted. It often looks at how research within the ESP field has progressed over time or how a topic has developed across research traditions (Snyder, 2019). Being cognizant of the history of ESP and how its roots have adapted to societal needs may provide a broader perspective on its past with implications for current significance, thus allowing us to have a better grasp of current trends and concepts related to ESP.

The first step was to scan the relevant selected literature in relation to the research agenda. In total, aside from physical book and journal article references accessed mainly through libraries, the keywords “ESP development,” “Needs Analysis,” “future of ESP,” “technology of ESP” and “interculture and ESP” were keyed into Web of Science (WOS), Google Scholar and a website in China—the National Knowledge Infrastructure (CNKI). More than 400 articles out of 1000 of articles, books and reports related to ESP were finally downloaded. They were most relevant to the research and contained the highest number of recorded citations. The MarginNote 3 app from Apple program helps to scan all literature with notetaking, summary facilities, which include the drawing of mind maps and the different topics were then drawn together to provide a coherent literature review. Endnotes app also helps the establishing of inclusion and exclusion criteria that can trim the body of ESP literature, such as, year of publication, the language of articles (English or Chinese), and type of article and journal. These procedures resulted in careful data extraction to ensure quality and validity in the review process. In compiling the data, due attention was given to author information, especially when attending to seminal theories, and their arguments. A comparative method ensued to identify similarities and differences in their arguments in order to construct a meaningful and discerning flow of the discourse. In addition, the review also considered the currency of the writings, which is important as a benchmark for updated literature on a research issue. On another note, the researchers considered the reputation of the publishers and journals, and perspectives were sought from different countries to pan the diversity and breadth of the relevant work done. The literature review data were then organized according to the five keyword topics and the information was then captured in a number of specific literature matrices that were tailored to the selected topic or theme that would help in the final reporting. As a result of the LR process, the final outline for the literature review report evolved and it then served as a guide for the drafting of the report and the finalizing of the selected bibliographic entries.

## 3. Historical overview of ESP development

Following the LR methodological procedure, the first theme that emerged was the historical overview of ESP development which traces the origin and history of ESP. In this review, each phase of development since ESP gained traction in the 1960s was noted. The historical overview will be presented and described according to the traditional four phases of ESP history identified by Johns (2012). Occasionally, noticeable overlaps were recorded as expected, as changes do not happen in isolation but usually they conflate and interact (Ramírez, 2015). Admittedly, this panning of ESP literature on its development is not entirely novel. However, each LR on an identified area could lend new insights, particularly when viewed from the lenses of current literature updates as a dynamic endeavor which could incorporate layers of newer viewpoints into an established field. Along with the chronological tracing, this review also provides insights into the application of specific linguistic and non-linguistic concepts in ESP teaching that could contribute to new emerging trends in the state of the art.

### 3.1. Early ESP development from 1962 to 1981

According to their landmark work, Hutchinson and Waters (1987) identified three factors that had a bearing on driving ESP development in the early years. They are: (1) the demands of the “Brave New World,” (2) a linguistic revolution, and (3) a newfound focus on the learner.

Demands of the “Brave New World” that impacted the origins of ESP can be traced back to the conclusion of the Second World War when people began to recognize the necessity of learning English (widely regarded as a global language) in the new commerce-driven world. The end of the Second World War had ushered in an era of tremendous expansion in scientific, technical, and economic activities worldwide due mainly to the growing economic strength of America. These changes created new and dynamic roles in the use of the English language leading to its consolidation as a significant international language. Greater impetus for its development is the Oil Crisis in the early 1970s which resulted in the flow of Western currency and knowledge into oil-rich countries. With these developments, new demands were made on the learning of the English language, thus impacting the growth of ESP with discernible teaching trends. Non-native speakers had to be equipped with a new *lingua franca* rooted in ESP that met their needs for cross-cultural communication, commercial transactions, and information exchange (Teodorescu, 2010).

#### 3.1.1. The teaching of functional vocabulary

The afore-mentioned events, and subsequent growth in ESP placed strong pressure on the language teaching profession to produce fast track results in the 1970s. In this regard, linguists promoted register analysis to enhance the development of scientific and technical lexis which was hypothesized to give ESP learning a boost. ESP practitioners, in turn, identified their major role as imparting and training of technical vocabulary related to a field of study or profession. For example, if one were educating nursing students, his/her job would be to teach them nursing terminology in a designed curriculum that was “flavoured” with medical and nursing terms (Johns, 2012).

At the same time, the learning of English for Speakers of Other Languages (ESOL) impacted the move toward learner-centered teaching. This movement resulted in great emphasis on learner needs and needs analysis as the foundation of ESP course design. Another major outcome, as stated by Hutchinson and Waters (1987), is the realization that marked differences in language use exist depending on the language skills learnt. To illustrate, language use can be determined by whether learners are learning to write or speak in the acquiring of language skills. In other words, the context of use will effect the needs of the learner. As a result, several attempts to refine and define language use for Science and Technology (West, 1994) materialized in the late 1960s and early 1970s. EST Pioneers like Selinker, Swales, Ewer, Latorre and Trimble were the trend setters. Notably, Swales (2004) added that “new” ESP teachers should begin to teach general English infused with technical vocabulary based on the needs analysis protocols that identified details about the language skill use to accompany the learners’ discipline. Thus, specific skill-based courses were popularly designed to meet learners’ specific foreign language needs based on needs analysis. For example, in English for Business Purposes, due consideration is given to the needs to the specifics of Business Writing, Making Business Presentations, or Reading Skills for Business. However, it soon became clear that simply teaching according to specific skills is an inadequate approach; rather, learners’ language-learning processes must also be addressed to enable successful real-life language learning. As a result, learners’ motivation and strategy use for mastering the target language were focused on, alongside needs analysis (Maleki, 2008).

#### 3.1.2. ESP teaching influenced by rhetorical and discourse analysis

Register analysis then gave way to the application of discourse analysis to obtain a better understanding of ESP discourse for teaching. At this point of time, register analysis was criticized as giving excessive focus on language form, and had failed to explain why and how sentences were created and integrated for use in specific disciplinary situations. Rhetorical and discourse analysis then took center stage as the emergent teaching trend and it “introduced the notion of linking language form to language use, making use of the key criterion for the selection of ESP teaching materials” (Dudley-Evans and Saint John, 1998, p. 22). The emphasis of discourse analysis is said to manifest the “communicative values” of a meaningful discourse. Hence, the ESP scope had expanded to a concentration on text analysis in association with discourse analysis.

Contrastive rhetoric, focusing on contrastive discourse analysis, has also always remained firmly linked to ESP research (Connor et al., 2002). For example, Swales and Mustafa (1984) published an earlier collection of works on the topic which featured the chapter of Holes (1984) entitled “Textual approximation in the teaching of academic writing to Arab students: A contrastive approach.” Additionally, Salager-Meyer’s (1990) comparison of metaphors found in French, Spanish and English medical research publications was published in the *ESPJ* in 1990. This interest in international rhetoric was alive and well at that time, especially in ESL development (Escudero and Swales, 2011). Bhatia et al. (2011) through their publications, contributed important intercultural volumes on legal discourse.

### 3.2. ESP development from 1981 to the 1990s

ESP development from 1981 to the 1990s took on a new significance as a result of the rapid dissemination of published ESP work. Hewings (2002) investigated the *English for Specific Purposes Journal* (ESPJ) publications and concluded that the journal had published a noticeable number of ESP studies outside of the United States and the United Kingdom, which are traditional sources of ESP work. The deep interest in ESP had spun over countries, such as, China, Central America, and South America, and Hong Kong as ESP hubs for information dissemination. He further concluded that there was a growing wave of recognition of ESP as an academic discipline internationally. Another finding was the diversity of ESP topic coverage that included development in EAP and EOP with their many sub-branches. Thirdly, as a result of these developments, general English as the more generic approach to program design seemed to be given less emphasis. Instead, more emphasis was placed on the learning of ESP with discourse analysis as a methodological procedure. In addition, ESP practitioners appeared to concern themselves substantially with specific target settings in language learning, and giving due regard to the design and production of ESP-oriented materials to enhance the development of ESP courses (Hewings, 2002).

Along with the show of deep interest in ESP research, the journal added many special issues targeted at specific learning to further equip practitioners with ESP teaching skills, covering domains ranging from ESP teacher training (Ewer, 1983), vocational ESP (Crandall and Burkart, 1984), interlanguage (Selinker and Douglas, 1987), to international teaching assistant training (Young, 1989). In the regular issues, three most frequently published themes throughout this period were reflected in: (a) *Needs Assessment/Needs Analysis and Learning-centered Approach*, (b) the *Wide-angle Approach* vs. *the Narrow-angle Approach in ESP* and (c) *Genre Analysis and Rhetorical Moves.*

The discussions thus generated a consolidated platform for ESP debate and argument. To illustrate, Jacobson (1986) focused on the strategic language learning goals of students working in a physics lab, while Tarantino (1988) provided face-to-face questionnaire interview data from 53 EST researchers and students to reveal how macro-and micro-level language requirements were assessed. Later, West (1994) demonstrated how research on the complexity of student needs analysis could become more empirical in the making.

Other scholars also contributed their seminal treatises in the form of books which also accelerated the ESP movement. Among them, Hutchinson and Waters (1987) initiated a major reexamination of many long-held ESP beliefs, such as, the issue of ESP students being guided toward developing “underlying competence” so as to become independent learners. Hutchinson and Waters’s (1987) work led on to further ESP developments that related to the learner-centered approach which “focuses on the learning process, emphasizes the exploitation of the learner’s already possessed skills (learned at work or *via* academic study), and takes into consideration students’ various learning styles” (Dudley-Evans and Saint John, 1998, p. 25). As a result, focusing on the needs of learners is just as important as the methods used to disseminate linguistic information and to address the salient affective factors that accompany the learning. In fact, “learner-centred” and “learning-centred” are still catchwords in ESP learning today.

#### 3.2.1. Teaching ESP in the 2nd phase of ESP development (1981–1990s)

Greater details on how best to teach ESP emerged from the discussion on the use of the Wide-angle Approach vs. The Narrow-angle Approach in learning orientations. The former advocates the teaching of English through topics beyond students’ specialist areas, while the latter focuses on students’ specific area of development (Johns, 2012). As Dudley-Evans and Saint John (1998) saw it, ESP theoretical work tends to lag behind practical material development at this period of time. The on-going argument about learning approaches also led to the questioning of the inclusion of an ESP subject specialist, who can give the necessary disciplinary content knowledge and applied language use input to the teaching environment. Both Tarone et al. (1981) and Johns (2012) strongly advocated this input leading to “subject-specialist informants” being prominently used in ESP teaching and research. In sum, the 1980s saw the trending of content and skill specificity, material design, and the use of the expert informant as facilitator or instructor as salient points of discussion in ESP development (Ramírez, 2015).

#### 3.2.2. ESP learning influenced by genre analysis and rhetorical moves

Another significant contributor to ESP development in the 1980s, was the growing influence of two new twin key concepts, that of “genre analysis” and “rhetorical moves” which became the bedrock of substantial ESP research (Johns, 2012). Toward this end, she clearly reinforced Paltridge and Starfield’s (2011) earlier advocacy on the need to further understand the discourse features of ESP texts if ESP learning is to be improved:

*[…] in drawing a clear distinction between research and practice, unlike many other research areas in theoretical and applied linguistics, ESP has been, at its core, a practitioners’ movement, devoted to establishing, through careful research, the needs and relevant discourse features for a targeted group of students* (Johns, 2012, *p. 6*).

The seminal article of Swales (2011), “*Aspects of Article Introductions*” and his books on genre analysis further perpetuated the importance of genre analysis and rhetorical moves in ESP research. However, in this phase of ESP development, most ESP research had remained more focused on EAP, especially EST at the post-secondary or graduate level for ESP learning. Also written discourse remained the preferred data for analysis (Hewings, 2002). As for its influence on learning approaches, the application of such ESP research information was noted to be ostensibly slow at the classroom level. A possible reason could be that the practitioners took time to take on the challenge, especially when the concepts may not be easily translated into teaching materials. On top of it, writing skills are usually considered more complex and difficult to learn compared to other language skills.

### 3.3. The modern age in ESP development (1990–2011)

During this period, more interesting and vibrant developments took place in the ESP arena. Among them were the flourishing of new ESP oriented work that focus on intercultural rhetoric, genre studies and corpus studies as evidenced in publications in new journals in the field. During this era, two new international journals were launched and considered on par to *ESPJ* for ESP work. Leki and Silva (1992) launched *the Journal of Second Language Writing* (JSLW), while Hamp-Lyons and Lumley (2001) founded *the Journal of English for Academic Purposes* (JEAP) in response to the overwhelming number of ESP related papers submitted for publication in the ESPJ. Paltridge and Starfield (2011) analyzed the publications in the ESPJ for 2010 and the breakdown revealed the following figures. Taiwan scored the highest number of submissions (23), followed by China (21). The United States recorded (16), Iran (11), and Malaysia (10). However, the acceptance rate for the *ESPJ* was only around 25%, indicating that a significant portion of submissions was not published, and there was a definite need for more publishing opportunities for ESP explorations.

Another significant feature of the 1990 to 2011 ESP era is the dominance of genre-focused ESP research, which tied up with Swales and Swales’ (1990) *Genre Analysis,* sparking off a highly fruitful debate among academics (Johns, 2012). Advanced academic genre studies continued to dominate, as demonstrated through works by Crandall and Burkart (1984) and Bhatia et al. (2011) on legal discourse. Interest in expository genres also enhanced research both within the ESP community and among genre theorists and practitioners from various theoretical schools (Bawarshi and Reiff, 2011).

While genre analysis gained traction, it was a source of concern for other scholars as they felt that the most intractable academic difficulties are still found in novice undergraduate language education (Benesch, 1996; Johns, 2012). Thus, outcomes of genre analysis may not be very useful when applied to fundamental learning of the English language.

#### 3.3.1. Continuing influence of genre analysis on ESP teaching and corpus analysis

Nonetheless, genre analysis continued to flourish and Hyon (1996) forwarded three recognized theories and approaches in genre studies: ESP, North American New Rhetoric studies, and Australian systemic functional linguistics, each reflecting viewpoints and educational aims that often contradicted one another (Johns et al., 2006; Tardy, 2012). Some other specialists (Johns et al., 2006; Swales, 2009; Flowerdew, 2011; Johns, 2012) worked on theory, research, and pedagogical convergences to address some of the theoretical and pedagogical discrepancies, which was projected as extremely helpful for future ESP research.

Swales and Swales (1990) continue to provide new angles to the analysis of a genre. In “*Other Floors, Other Voices*,” Swales (1998) brought photography into the ESP lexicon, as he studied the interactions of texts and contexts in three distinct discourse communities using written work, interviews, and observations. Swales (2004) coined a specific novel phrase, “occluded genres,” which perform “essential way stage roles in the administrative and evaluative functioning of the research worlds” (p. 18). Paltridge and Starfield (2011), who are active ESP practitioners and researchers, expanded the photography element in genre studies in their more current work.

The modern period of ESP development was characterized by another research innovation that uses corpus research, notably in studies of written academic genres. During this time, the relevance of inter-personality and interactivity in written discourse grew to the point where one aspect, evaluative language, became a focal point for discussion as evidenced in a 2003 special issue of the *JEAP* (*Journal of English for Academic Purposes*) on “evaluation in academic discourse.” Hunston and Thompson (2000) in their contribution, defined evaluative language as the “expression of the speaker or writer’s attitude or stance toward, or viewpoint on, our feelings about the entities or propositions that he or she is talking about” (p. 5). Within and across academic discourses, the contributors to this issue explored critically what evaluative language is and its purposes (Johns, 2012). Indeed, Johns (2012) saw the flourishing of global ESP journals as playing a major role in articulating the importance of ESP and its relevance in English language development. Furthermore, the increased emphasis on well-established concepts such as international rhetoric and genre analysis research, along with the more in-depth and continuing research on corpus studies, continued to demonstrate their presence as anchored core issues, with room yet for the forging forward of evolving growth in the field.

### 3.4. Future vistas in post-modern ESP development and teaching

Following the three marked phases in ESP historical development and teaching, it would be apt to consider what lies beyond the phases as future vistas in ESP development. The early developments to the current 21st century have seen a deep-seated entrenchment of ESP teaching often aligned to the identification of needs. As such, the following section on future vistas will explore specifically the changes in defining needs analysis and its implications for teaching while noting the theoretical inputs that have contributed to ESP practice.

#### 3.4.1. Needs analysis revisited

Needs analysis or needs assessment often forms the corner stone of ESP practice and it is envisaged to continue to be so in future ESP works. Since its appearance in the 1970s, needs analysis has evolved as a framework that has attracted and garnered much attention of many ESP and ESL researchers and practitioners. Among the influential works are those by Munby (1978), Hutchinson and Waters (1987), Robinson (1991), West (1994), Dudley-Evans and Saint John (1998), and Iwai et al. (1999) who explicated the role and importance of needs analysis not only for ESP courses but also for those that concern English for General Purposes. Strevens (1977) also emphasized that needs analysis, which is primarily concerned with the character of scientific discourse, is a necessary first step in ESP course formulation. Richterich and Chancerel (1978) and Trim (1978) extended the dimensions of needs analysis to include the full range of techniques that can be tapped to lead to an understanding of the parameters for successful learning, such as the ego of learners, teachers, administrators, course-writers, and producers, expectations of careers and workplace satisfaction, social dynamics, learner type, and resource analysis. Needs assessment, according to Holec (1980), Hutchinson and Waters (1987), and Hamp-Lyons and Lumley (2001), is a classic process for establishing a close link between learners and curricula. Needs analysis determines learners’ needs and helps prioritize the setting of learning targets that enables efficient language teaching.

The two intertwined concepts of “needs” and “analysis” are defined in terms of necessities, wants, or lacks (Hutchinson and Waters, 1987). These “needs” may vary depending on “the goal of analysis but both focus on the learner, and the manifestations can be described as objective or subjective, perceived or felt” (Brindley, 1989). Thus, meeting objective and subjective needs would depend largely on target situation analysis in which learners’ real-world communicative needs are recognized in order to set some founding principles for course design.

As for subjective needs, Nunan et al. (1988) commented:

*Subjective needs […] are situated in the present and influence the teaching methodology of the syllabus, taking into account biographical information and learner preferences, thus going into why the learner wants to learn a foreign language and the classroom tasks and activities that the learners prefer (p. 18).*

Nunan et al. (1988) together agreed that needs analysis is the key aspect of curriculum development and is typically imperative before a syllabus for language teaching can be prepared.

In a nutshell, Robinson (1991) believed that “Needs analysis is usually considered as fundamental to ESP, but ESP is by no means the only educational endeavor that uses it,” (p. 7). Alternatively, West (1994) defined needs as what learners will need to do with the foreign language in the target learning situation, and how learners might best master the language throughout the learning period. These needs may then be translated as program’s goals and objectives to serve as the foundation for the development and selection of teaching materials and learning activities, besides aiding test development and evaluation of learning strategies.

Along similar lines, Brown (1995) argued that:

*Needs analysis is the systematic collection and analysis of all subjective and objective information necessary to define and validate defensible curriculum purposes that satisfy the language learning requirements of students within the context of the particular institutions that influence the learning and teaching situation (p. 102).*

The articulated needs set the target learning goals and are reflected in the defining of language use governed by the context for learning, realized in specific disciplines, such as for academic purposes, business, sports or aviation. In future, it is likely that more novel ESP courses will arise due to the evolving changes in disciplinary needs, whether objective or subjective. In the event, relevant materials can be designed. Information from discourse and genre analysis can help feed meaningful analyses of the needs. Objective needs can also be generated from the needs of the location of study or workplace and its demands compounded by the social situation, thereby adding to the complexity of the “needs” concept.

While basic notions about needs analysis remain relatively constant over the years, however, Otilia and Brancusi (2015) observed the concept of needs analysis has undergone changes. They noted that in the early stages of ESP development (i.e., the 1960s and early 1970s), needs analysis basically revolves around assessing learners’ communicative needs and identifying the techniques required to achieve appropriate teaching objectives. Today, the objectives of needs analysis are considerably more complicated, aiming to gather information from many dimensions of students’ experience that can fall within the objective context as well the ESP learning environment. Abuklaish (2014) added that data can be gathered from questionnaires, tests, interviews, and observations to discover the following:

- The situations in which a language will be used (including who it will be used with).
- The objectives and purposes for which the language is needed.
- The type of communication that will be used (e.g., written, spoken, formal, informal).
- The level of proficiency that will be required (p. 51).

Dudley-Evans and Saint John (1998) in their book, addressed the original definition of needs analysis as being related to “ESP (as) a discipline that attempts to meet the needs of a specific population of students, employs methodologies and material from the discipline it is centered on and focuses on the language and discourse related to it” (p. 125). Graves and Xu (2000) has redefined it as a system that entails “a systematic and ongoing process of gathering information about students’ needs and preferences, interpreting the information, and then making course decisions based on the interpretation to meet the needs” (p. 98). This updated definition views needs analysis as a dynamic process dotted with several important decision-making steps.

Braine (2001) concluded from previous descriptions of needs analysis that disagreements among linguists on its specific meaning as not uncommon, though they all concur on underlying external factors like staffing, time, and cultural attitudes that influence the multiple definitions. Emphasizing procedural aspects, Hyland (2006, p. 73) had seen needs analysis as “the procedures involved in data acquisition as the framework for the analysis, which may then be prioritized, of the needs of students or groups.” The procedures entailed looking into the following: (1) learners’ goals and backgrounds, (2) their language learning preferences, and (3) the situations in which they will need to communicate, which may determine what learners know, do not know, or want to know, and can be collected and analyzed in a variety of methods. Notably, these processes today should not be a one-time event but should be continuously refined alongside the development of ESP teaching and learning. Regardless of the varied definitions, all learners and relevant members of the community should be involved participants in the meaningful planning process.

##### 3.4.1.1. Recent related studies on ESP and needs analysis

In support of ESP work that has evolved as multi-dimensional in approach, which is still developing, some current sample readings are explored to provide more insights. For example, Chen et al. (2019) embarked on the use of needs analysis which employed triangulated data gathered from questionnaires, semi-structured interviews, and on-site observations. Based on the data obtained, they built a case for a context-aware ubiquitous learning environment. Their findings revealed that language learning should be done in the following sequence: listening, reading, and speaking skills, followed by green-building terminology. Video and audio content, adaptive functionalities, and a user-friendly interface design were also found to be necessary to promote successful learning.

Pazoki and Alemi (2020) explored learner attitude with regard to ESP learning in Iran. Results indicated that the students had negative attitudes toward ESP learning experiences, and this was linked to a low motivation level. They emphasized the importance of doing a reliable needs analysis so that stakeholders are more accurate in defining needs for goal formulation and avoiding discrepancies in course material selection. Students’ motivation in ESP classes could also be subjected to ongoing evaluation at various planned learning stages as a tracer effort in monitoring progress. At the macro level, problems may be related to policymaking. Thus, stakeholders must take this into consideration to set realistic learning goals.

Identifying students’ needs ultimately requires a thorough investigation of many elements. Tailoring a course to meet the needs of the learners in and of itself does not ensure course success and effectiveness. Motivation to learn plays a crucial role in ensuring learning success. Pazoki and Alemi (2020) emphasized that a well-designed course could capture the interest of learners, and this could increase the level of motivation for attaining success in learning the language.

On the other hand, Arnó-Macià et al. (2020) reported that their students are generally satisfied with their ESP classes and had gained a much better understanding of the nature of specialized communication. The findings also provided deeper insights into students’ strategy use and in areas where ESP was useful in preparing and empowering the students for their future workplace. The findings can be used to inform on the success of needs analysis that will further assist the design of other ESP courses.

ESP teachers may need to help learners to attend to general academic needs as the immediate goal. Academic fulfillment like reporting on project work and completing course assignments in English is just as important as fulfilling professional or workplace-related needs like writing professional emails, attending job interviews, and doing specialist oral presentations. In this way, rather than depending on intuition, ESP teachers may develop courses based on information obtained through personalized needs analysis. Indeed, support given to academic fulfillment as part of a well-designed ESP course may boost learners’ confidence, fluency, and accuracy as they enter into specialized communication. In an increasingly internationalized context, it is also necessary to reappraise current ESP courses and practices to determine the extent to which they are adapted to the ever-changing needs in a globalized world.

In Indonesia, Sari and Sari (2020) used a qualitative descriptive method to investigate the English language needs of marine pilots. Their study revealed the real-time needs of this group of language users, with a focus on practical language skills (speaking and listening) skewed to giving advice and exchanging information. In ESP learning, they emphasized that it is important to provide learners with a meaningful learning experience, and the use of role-play and simulation could go a long way in achieving communicative competence.

In Malaysia, Shaalan (2020) conducted a needs analysis on dental majors to assess students’ academic and professional needs, wants and lacks for the designing of a course that fits their requirements. The findings indicated that incorporating project-based learning techniques into the ESP course helped to increase dental vocabulary and using creative techniques in the dental vocabulary classroom boosted students’ self-awareness, problem-solving skills, critical thinking, and creativity.

College English is a mandatory course for students who enter tertiary education in China. The exit language performance indicator is a set criterion score obtained through the CET (College English Test) as a graduating language assessment attainment goal. This requirement is often viewed as a less than satisfactory rigid language policy implementation. The tension between ESP approaches and College English which centers on the learning of general English has been much debated among Chinese scholars. Xu and Liu (2015) point out that “after students have reached the level of general requirements in the English curriculum, they can gradually transit to ESP” as the next step, thereby recommending an integrated approach as the way forward in the learning of English. This call is supported by Liu (2003) who views ESP teaching as “an extension and continuation of basic English teaching” with Wen (2014) advocating a similar line of action on a symbiotic co-existence of EGP and ESP in the university English teaching system. In a series of papers, Cai (2007, 2010, 2015) repeatedly emphasizes that the difference between EAP and EGP (English for general purposes) lies merely in goal identification and suggests that universities could replace the integrated EGP course with academic English as a starter course for the freshmen year as a more realistic learning goal in the EFL context. This recommendation could be aligned to the provision of ESP as an academic English course and could be seen as a possible path for students of any discipline to engage themselves in a more meaningful manner in their English language learning experience. The premise taken is that after an ESP course that is oriented toward academic English, it could motivate them to study ESP further to equip themselves with more marketable skills within their disciplines when they leave the university.

Attesting that ESP research in China has developed rapidly in recent decades, Lu and Jiang (2018) analyzed ESP research studies published from 2007 to 2016 (a period of 10 years) in China’s core online journal database, CNKI (China National Knowledge Infrastructure). The results revealed that China’s ESP research studies have been on the rise in the 10 years surveyed, and the research areas have expanded from non-empirical studies with a concentrate on theoretical reflections (occupying 80.3% of the total publications) to empirical studies (qualitative research, qualitative research, and mixed methods research) standing at 19.7%. Among the empirical research, needs analysis only constituted 10.4% of the pool of studies. These figures illustrate a dire lack of ESP research in China and local ESP researchers could definitely play a vital role in adding insights and understanding into the needs of EFL learners in relation to the target language use settings, with a possible greater inclusion of learning ESP oriented courses designed and developed for individual group of learners.

In brief, various current studies have been found to identify learners’ specific needs (Long, 2005; Chaudron et al., 2010; Spence and Liu, 2013; Chen et al., 2019). In addition, Mirisaee and Zin (2009) recommended the use of triangulated data to develop effective task-based instruction. In practice, a successful needs analysis should look at essential language skill needs from a variety of angles. Following the needs that are discovered, a subsequent step is to use the information to develop effective ESP materials (McDonough, 2010).

#### 3.4.2. Use of information technology

From the above description, ESP development is certainly affected by ever-changing needs and contextual challenges. Future vistas about ESP developments and learning potentially can result in a more flourishing and thriving field. Lesiak-Bielawska (2015) aptly noted that by the turn of the 20th century, technology noticeably has impacted foreign language learning. Since then, there had been a deluge of such developments that made the Internet a source of diverse technological applications. However, she cautioned that much more research is necessary as she felt that many gaps existed. For one, she questioned the sustainability of some technologies as they may become obsolete and be replaced by new innovations. ESP teachers may have a hard time “catching up” as many are “digital immigrants” and the road to being “digital natives” could be fraught with many challenges.

Nonetheless, it is a fact that technology is here to stay and it is also an ever-growing concern as more and more transborder transactions take place. With the world experiencing fast-paced technological advances, it is inevitable that it will impact current and future language use. Technology can be said to be perversive especially in the workforce. Every country bent on economic growth must attend to advances in technology so as to remain competitive. These national needs are translated into the realm of language teaching.

##### 3.4.2.1. Recent related studies on ESP and information technology

As a line of investigation, Asmali (2018) purports that integrating technology in an ESP curriculum will be highly beneficial to students as computer technology allows interactive and communicative activities that can be professionally related to the current or future workplace. Through specially designed multimedia packages tailored to students’ needs, students can easily be exposed to authentic target language use, thus allowing more language practice that is often lacking in traditional materials.

Yan and Li (2018) pointed out that big data, cloud computing, and information sharing can be combined through a triangulation analysis to generate more precise needs analysis relevant to the underlying concepts of Present Situation Analysis, Learners’ Needs Analysis and Target Situation Analysis. In line with these current developments, new ESP approaches that incorporate technology inevitably will need to be devised and taught to post-modern ESP participants, especially professionals who are in need for tailored ESP contact to accelerate professional advancement.

With specific needs analysis results, teaching materials could definitely be designed for a specific group of individuals. During the teaching process, simulated teaching methods, task-based teaching methods, cooperative project-based learning (Zaafour and Salaberri-Ramiro, 2022) and flipped methods might guide the teaching application to meet the learners’ needs. In addition, ESP teachers can resort to blended learning (Salim et al., 2018) or the design of a Massive Open Online Course (MOOC; Shapiro et al., 2017), use other information and communication technology tools to design multimodal online platforms (Yélamos-Guerra et al., 2022). The use of technology is well illustrated in the book, *English in the Disciplines: A Multidimensional Model for ESP Course Design* in which the authors, Hafner and Miller (2019) stated their concern for multi-literacies and the greater need for collaborative learning. They highlighted the use of ESP interactive collaborative learning through the use of LearnWeb 2.0 and corpus analysis, and then proceeded to present illustrative scenarios in an ESP course that learners can immerse in to become multi-literate and gain useful professional knowledge and critical application.

In China, learners can access technical support for English learning *via* the ESP Linguistic Corpus (Zheng and Wu, 2019) and Zhu (2020) highlighted the application of e-portfolios and apps to set up ESP competency standards enabling the establishment of an evaluation test system to standardize ESP language assessment mechanism. All these could well be future characteristics in ESP teaching and learning and recognizably pave another significant ESP phase of “a brave new world.” These implementations would provide more information for the future growth of ESP research as well as enhancement of ESP subjective status, at the same time, boosting practical development of ESP-oriented courses (Cai, 2019). Technology without a doubt is making a great impact on ESP and will continue to do so in the near future.

#### 3.4.3. Intercultural communication

Lesiak-Bielawska (2015) also noted when she shared as a “*second wave of online learning*,” the importance of cultural learning that may also connect to intercultural competence, cultural literacy and social discourses. With regard to English as a *lingua franca* in an EFL situation, Firth (1996) defined cultural learning as “a contact language between persons who share neither a common native tongue nor a common (national) culture” (p. 240). In this context, cultural awareness in both native speakers’ culture and non-mainstream culture should be seriously considered in the teaching of English to non-native speakers.

##### 3.4.3.1. Recent related studies on ESP and intercultural communication

Translingual efficiency could stress the need for effective negotiation strategies when communicating across linguistic and cultural boundaries (McIntosh et al., 2017). Thus, intercultural communication and awareness within linguistic diversity is a needed mainstay component in English learning classrooms (Jiang et al., 2020) inclusive of ESP classrooms by extension. As pointed out by Benabdallah and Belmikki (2020), learning a language does not occur in a vacuum, but rather it is carried in social, economic and cultural contexts so that learning becomes meaningful. An ESP-oriented course especially in an intercultural context, could also call upon such needs to design suitable materials for vocational activities to enhance ESP linguistic and cultural competency, pertinently determined for workplace demands (Hazrati, 2014).

This is well-illustrated in English courses, targeted specifically in areas, such as, shipping (Bocanegra-Valle, 2014), corporate leader messages in business (Ngai et al., 2020), investor relations communication financial policy (Camiciottoli, 2020), advanced business writing (Feng and Du-Babcock, 2016), and aviation English (Hazrati, 2014).

It is also important to distinguish between Culture with a “big C” (inclusive of history, geography, institutions, literature, art and music, etc.) and with a little “c” (e.g., cultural behavior, culturally influenced beliefs, and perceptions). In the designing of materials, Connor and Traversa (2014) believed that the “little” culture is more linked to language learning to promote cohesive behaviors within social groupings, and she recommended incorporating five complex and interacting small cultures for teaching: national culture, professional-academic culture, classroom culture, student culture and youth culture (Xu et al., 2016). Indeed, the intercultural rhetoric framework has made valuable pedagogical contributions to the teaching of EAP/ESP with a recognition given to societal and student needs, teacher preparation, and curriculum design in particular contexts (Xu et al., 2016; McIntosh et al., 2017).

From another perspective, Yang and Wyatt (2021) asserted the crucial role of cultural influences on improving Chinese ESP teachers’ cognitions about motivation and motivational practices, with suggestions on how the Confucius heritage, as a deep-seated Chinese cultural feature, can be conceptually applied to ESP courses for East Asian countries as a point of cross-cultural awareness. Learning English without understanding its cultural context is a definite risk (Alharbi, 2022), and as a future vista, this concern should continue to pervade in the application of an ESP pedagogical framework.

## 4. Conclusion

This review study began by introducing the basic tenets and trends of ESP giving a historical account of ESP development which was accompanied by a discussion of how ESP teaching and learning have evolved concurrently. This recognition of history sets the tone for possible future trends in ESP development and teaching giving due emphasis on the need for a revisit of needs analysis, the salience of technological advances, and intercultural communication. In sum, new explorations in needs analysis, technological advances, and intercultural awareness will significantly support the construction of a better environment for learning English in specific situations, therefore justifying the establishment of future ESP courses to properly prepare students and train professionals for the workplace. To confirm the effectiveness of the use of ESP frameworks, it is reiterated that it is equally vital to recognize that changes in ESP development will also result in changes in learning approaches that must be taken seriously into account for successful learning.

## Author contributions

AD, SC, and MW contributed to the conception and design of the study. AD wrote the first draft of the manuscript. SC and MW revised sections of the manuscript. All authors contributed to the article and approved the submitted version.

## Conflict of interest

The authors declare that the research was conducted in the absence of any commercial or financial relationships that could be construed as a potential conflict of interest.

## Publisher’s note

All claims expressed in this article are solely those of the authors and do not necessarily represent those of their affiliated organizations, or those of the publisher, the editors and the reviewers. Any product that may be evaluated in this article, or claim that may be made by its manufacturer, is not guaranteed or endorsed by the publisher.
